# Signal detection of substance-related emergency department visits during UEFA EURO 2024 in Germany: enhancing public health surveillance for future mass gatherings

**DOI:** 10.3389/fpubh.2026.1876317

**Published:** 2026-10-05

**Authors:** Carmen Schlump, Madlen Schranz, Nesrine Charfeddine, Mirjam Rupprecht, Alexander Ullrich, Michaela Diercke, AKTIN Research Group, Caroline Grupp, Bernhard Kumle, Bernadett Erdmann, Conrad Grehn, André Gries, Eric Handmann, Domagoj Schunk, Christoph Lewejohann, Matthias Nürnberger, Philipp Kümpers, Stephan Arlt, Harald Dormann, Marina Karg, Martin Bergold, Thomas Henke, Bernd Ulrich, Christian Reich, Andreas Hauf, Thomas Peschel, Thomas Peschel, Thomas Peschel, Kirsten Habbinga, Norbert Pfeufer, Heike Höger-Schmidt, Thomas Baitz, Jörg Brokmann, Carsten Mach, Rupert Grashey, Martin Möckel, Ralf Offermann, Anna Slagman, Rajan Somasundaram, Eike-Christian Kühn, Martin Möckel, Ralf Offermann, Anna Slagman, Tobias Hofmann, Susanne Drynda, Matthias Klein, Matthias Klein, Thomas Händl, Christian Wrede, Markus Wehler, Jürgen Neubauer, Christian Dumpies, Johannes Jubitz, Fabienne Jacob, Henstedt-Ulzburg Sabine Blaschke-Steinbrecher, Katrin Esslinger, Sebastian Wolfrum, Tobias Schilling, Friedrich Reichert, Jacqueline Bauer, Stephan Höft, Stephanie Matis, Sabrina Heinrich, Markus Zimmermann, Frank Hanses, Volker Burst, Hans-Jörg Busch, Felix P. Hans, Jonas Bienzeisler, Hauke Heidemeyer, Alexander Kombeiz, Raphael Majeed, Rainer RÖhrig, Susanne Drynda, Saskia Gieraths, Ronny Otto, Wiebke Schirrmeister, Felix Walcher, Juan Estupinan Mendez

**Affiliations:** 1 Robert Koch Institute, Department for Infectious Disease Epidemiology, Berlin, Germany; 2 Charité–Universitätsmedizin Berlin, Corporate Member of Freie Universität Berlin and Humboldt, Universität zu Berlin, Institute of Public Health, Berlin, Germany; 3 Charité–Universitätsmedizin Berlin, Corporate Member of Freie Universität Berlin and Humboldt, Universität zu Berlin, Emergency and Acute Medicine (CVK, CCM), Berlin, Germany; 4 AKTIN Infrastructure and Emergency Department Data Registry, Aachen/Magdeburg, Germany

**Keywords:** healthcare utilization, health monitoring, mass gathering surveillance, non-communicable diseases, substance use disorders, syndromic surveillance

## Abstract

**Introduction:**

Germany hosted the men's UEFA European Championship (EURO 2024) in summer 2024, attracting millions of visitors across multiple cities. Mass gathering events (MGEs) can rapidly alter social environments which impact substance use behaviors, potentially increasing demand on local healthcare systems. In response, the German national public health institute (Robert Koch Institute, RKI) piloted substance-related indicators using routine emergency department (ED) data from the Emergency Department Data Registry throughout the tournament to explore their applicability for real-time emergency department surveillance.

**Methods:**

We aimed to monitor substance use patterns in ED visits during EURO 2024 by developing an umbrella syndrome definition for psychotropic substance intoxication (SATX) and two more specific syndrome definitions for acute alcohol intoxication (ATX) and substance intoxication other than alcohol (STX), based on diagnoses, chief complaints, and age criteria. Descriptive analyses compared identified cases during the tournament period (14 June−14 July 2024) with a reference period. Analyses were stratified by sex, age group, and triage level. A quasi-Poisson regression model was used to estimate expected values and 95% prediction intervals (95% PI) for each indicator during the tournament period. Signals were detected when observed case counts exceeded the upper threshold of the 95% PI.

**Results:**

Between 1 January 2018 and 9 September 2024, 5,602,933 visits from 39 EDs were included. Across both the tournament and reference periods, SATX, ATX, and STX cases were more common among males, particularly for ATX. Most SATX and ATX cases occurred among individuals aged 30–59 years, whereas STX cases were most frequent among those aged 20–39 years. Relative increases in triage level 4 cases were observed during the tournament period. During EURO 2024, signals exceeding the upper threshold of the 95% PI were detected for all indicators, including 16 SATX, three ATX, and 13 STX signals. The largest deviation was observed for SATX on 29 June 2024, with 127 observed cases compared to 74 expected cases.

**Conclusion:**

This study demonstrated that rapid changes in substance-related ED utilization during MGEs can be effectively monitored using the syndrome definitions and signal detection methods, whose application can support public health surveillance during future events.

## 1 Introduction

Germany hosted the 17^th^ edition of the men's UEFA European Championship (EURO 2024) from 14 June 2024 to 14 July 2024. Such large-scale sporting events are usually accompanied by a high influx of tourists, increased travel activity, and heightened media attention. The EURO 2024 was attended by 24 teams competing in 51 matches. Approximately 2.7 million people attended these matches in stadiums, with an additional 6.2 million visiting fan zones (1). Across the country, around 2,100 EURO 2024-related events were held, including about 940 public viewing events to enable accessibility for a broader audience (2, 3).

Hosting mass gathering events (MGEs) of this magnitude has implications for public health. In Germany, the use of substances such as alcohol, illicit drugs, cannabis, and psychoactive medications continues to pose a considerable burden of disease, placing significant strain on local healthcare systems (4) including emergency departments (EDs), often serving as the first point of contact. MGEs like the EURO 2024 can further intensify these existing pressures (5–7), as changes in the social and environmental context can rapidly alter consumption patterns, leading to an immediate shift in demand for emergency care services (8–10).

Sporting events foster a celebratory atmosphere that particularly promotes the social acceptance and availability of legal substances. Such contextual factors are well-established predictors of altered drinking behavior, including the risk of binge drinking (8, 11, 12). Additionally, the competitive character of sporting events is often followed by heightened emotional responses, including both elevated excitement and potential frustration (5, 13, 14). This combination of factors creates a social context in which individuals may exceed their usual limits. While rapid increases in substance-related emergencies alone place additional strain on already stretched healthcare resources due to increased length of stay and resource utilization (15), secondary health consequences of substance use, such as accidents, may further contribute to ED overcrowding and access block (16). Both anticipated and unexpected changes may also disproportionately affect vulnerable subgroups, such as underage individuals (17), those with pre-existing health conditions (18), or populations with limited access to healthcare (19), thereby exacerbating existing health disparities and contributing to increased morbidity and mortality.

Therefore, timely information on effects on public health during MGEs is essential for supporting stakeholder decision-making and guiding targeted interventions. Internationally, using routine data from EDs for syndromic surveillance has proven beneficial for rapid identification and understanding these dynamics of substance-related healthcare utilization (20–24). Existing systematic reviews highlight varying impacts of mass gatherings on ED visits (5, 6, 12). Some studies focusing on alcohol-related visits report significant increases, particularly on game days compared to non-game days, while others find no substantial changes during sporting events (12). However, during the EURO 2016 football tournament in Marseille, alcohol-related ED visits rose by 43%, indicating elevated heavy drinking compared to before and after the tournament (25). Similarly, increases in substance-related ED visits including alcohol were observed during the opening ceremonies of the 2012 Olympic and Paralympic Games in London (21, 26) while presentations related to illicit drug use spiked following the closing ceremony of the 2000 Olympic Games in Australia (27). These findings underscore the importance for a population-level surveillance of substance use patterns during MGEs, as their public health impact may extend across the host country through public viewing events and other related gatherings beyond the official event locations. Such surveillance can inform future prevention strategies, provides public reassurance in the absence of adverse health outcomes, and enhances preparedness to respond to rapidly changing healthcare demands, ultimately contributing to improved patient outcomes during large-scale events.

During the EURO 2024, the Robert Koch Institute (RKI) in Germany conducted intensified surveillance of infectious diseases using various data sources, including ED data from the Emergency Department Data Registry (EDDR) (28, 29). The ED syndromic surveillance system at RKI provides timely information on ED utilization from participating EDs in the EDDR, enabling continuous monitoring of trends in infectious diseases. Hosting the EURO 2024 presented a valuable opportunity to further explore the potential of ED surveillance during MGEs for monitoring non-communicable diseases (NCDs), by piloting substance-related indicators and exploring their applicability in a real-time surveillance scenario.

This study focuses on monitoring three substance use indicators in ED data during the EURO 2024: a broad umbrella indicator encompassing all substance-related ED visits, and two specific indicators distinguishing between cases of acute alcohol intoxication and those involving substances other than alcohol. Findings from intensified surveillance are presented using descriptive analyses of case frequencies and demographics during the tournament period in comparison to a reference period. Signal detection methods were applied to identify significant deviations in daily cases and among age groups across indicators, aiming to test their suitability for future MGEs.

## 2 Methods

### 2.1 Ethical considerations and data protection

This study was conducted in accordance with the ethical principles of the Declaration of Helsinki. Ethical approval was obtained from the Ethics Committee of the Medical Faculty of Otto von Guericke University and University Hospital Magdeburg for the use of data from the Emergency Department Data Registry for non-communicable diseases and events (approval number: 89/24). For this study, we received approval to pilot syndromic surveillance of substance-related indicators during the EURO 2024 from the AKTIN Data Use and Access Committee (Data Access Request ID 2019–003, 4th amendment). All EDs currently contributing data for RKI's ED syndromic surveillance were contacted via email through the AKTIN Infrastructure, and only those that consented to the use of their data for the purpose of exploring NCD indicators were included in the present study. Data from EDs that either declined participation or did not respond to the request were excluded from the analysis.

Using health data for research purposes or in the field of public health from the EDDR is governed by Articles 89 and 9 (2) of the General Data Protection Regulation (EU) 2016/679 (Europäische Datenschutz-Grundverordnung, EU-DSGVO) and § 22 or § 27 of the German Federal Data Protection Act (Bundesdatenschutzgesetz, BDSG), which provide the legal framework for the protection of personal data. The AKTIN data protection concept details the measures implemented to ensure adherence with this legal framework (30). Consequently, ED data transmitted to RKI were anonymized and individuals are not identifiable. Therefore, repeated visits by the same patient cannot be linked.

### 2.2 Data basis

Routine case-based ED data are transmitted to the RKI on a daily basis via the AKTIN Infrastructure (28), where they are processed through an automated surveillance pipeline and stored in an internal database. As of September 2024, daily data transfers to the RKI for syndromic surveillance reporting were available from 49 of the then 58 EDs participating in the EDDR, covering 10 federal states.

For each visit, patient-level information includes age group and sex (categorized as male, female, other), along with administrative data such as date and time of visit, ED affiliation, and disposition. Triage level is documented using either *Manchester Triage System* (MTS) (31) or *Emergency Severity Index* (ESI) (32). The main reason for ED utilization is recorded as a chief complaint selected from the *Canadian Emergency Department Information System Presenting Complaint List* (CEDIS-PCL) (33). Diagnoses are documented using the *German Modification of the International Classification of Diseases, 10th Revision* (ICD-10-GM) in accordance with the official German coding guidelines (34).

Data collection and transmission for outpatient ED cases are regulated by § 295 SGB V (German Social Code Book V-Statutory Health Insurance), and require at least one diagnosis to be coded according to ICD-10-GM, including information on diagnostic certainty (e.g., “V” for suspected, “G” for confirmed, “A” for ruled out, or “Z” for status post). Inpatient cases fall under § 301 SGB V and must include at least one admission diagnosis, with the option to report up to 20 diagnoses per case (35). As a result, multiple diagnoses can be assigned to a single visit, although only one chief complaint is documented per patient (36). To be eligible for transfer to the RKI, each entry must be assigned a unique visit ID and include complete data on sex, age, date and time of visit, and ED affiliation.

### 2.3 Syndrome definition

Three syndrome definitions were developed through a collaborative process involving ED chiefs from participating clinics in the EDDR, representatives of the AKTIN Network and epidemiologists from the RKI. Based on routinely collected information, including chief complaints, diagnoses and patient age, the syndrome definitions were created to classify substance-related ED visits. A previously established and validated syndrome definition for identifying cases of acute alcohol intoxication (ATX) was adopted from earlier work (37); this syndrome definition is based exclusively on ICD-10-GM coded diagnoses. Building on this, two additional syndrome definitions were developed: one focusing on substance-related visits other than alcohol (STX), also based solely on ICD-10-GM coded diagnoses, and a broader umbrella definition (SATX) combining both alcohol- and non-alcohol-related cases. The SATX definition aims to capture any ED visit indicative of psychotropic substance use, identified through either relevant CEDIS-PCL values or ICD-10-GM coded diagnoses, or a combination of both. The minimum age threshold of over 10 years was defined *a priori* for all three syndrome definitions, as substance-related ED visits in younger children may disproportionately include unintentional exposures, which are not the primary focus of this surveillance for intentional recreational use (38). The same threshold has been applied in previous studies to distinguish intentional substance use from accidental ingestion (39) (Supplement 1).

### 2.4 Descriptive analyses

Descriptive analyses were based on the relative proportion of cases identified by each syndrome definition among all eligible ED visits. To account for varying completeness of data reporting across participating EDs, the denominator was restricted to ED visits that met the inclusion criteria of the respective syndrome definition. Specifically, for SATX, only visits that provided at least one ICD-10-GM diagnosis code or a chief complaint and involved patients aged 10 years or older were included, as only these visits had the potential to be classified as SATX cases. In contrast, for ATX and STX, the denominator was restricted to visits involving patients aged 10 years or older with ICD-10-GM coded diagnoses only, as case identification for these syndrome definitions relied exclusively on diagnostic information.

The EURO 2024 tournament lasted 31 days, from 14 June 2024 to 14 July 2024, and is hereafter referred to as the “tournament period”. To enable comparability across days of the week and account for seasonal effects, a reference period was defined comprising a pre-tournament phase (17 May 2024–2 June 2024; 17 days) and a post-tournament phase (15–28 July 2024; 14 days), totaling 31 days as well. To ensure higher data completeness and comparability, this reference period was selected instead of the summer months of previous years, as not all EDs started contributing data at the same time point. This allowed for the calculation of both, absolute case counts and relative proportions when stratifying identified cases for each indicator by sex, age group, and triage level. The sex category “other” was excluded due to small case numbers in order to reduce risk of re-identification and to ensure meaningful comparison, as it may include entries reflecting diverse gender identities as well as unspecified entries where information on sex or gender was unavailable. Relative changes (Δ%) were calculated as the percentage difference between tournament and reference period proportions, using the reference period as the denominator.

### 2.5 Signal detection

To timely detect deviations in case numbers, an adapted version of the Farrington Flexible algorithm that incorporates day-of-the week effects was applied. This method uses a quasi-Poisson regression to calculate an expected value and a 95% prediction interval (95% PI), accounting for seasonal fluctuations, day-of-week patterns, long-term trends, and variation on the total number of ED visits per day. Expected values were estimated using ED data from the previous 5 years, excluding the 26 most recent weeks before 1 May 2024. An expected distribution of case numbers was calculated for each day from 1 May to 31 August 2024, providing a broader observation window before, during, and after the tournament. This provided context for interpreting subsequent deviations during the tournament.

Deviations from the expected value are referred to as “detected signals” when observed case counts exceeded the upper threshold of the prediction interval (Supplement 2). Signal detection was applied to daily relative proportions of cases among eligible ED visits aggregated across all participating EDs (overall signals), as well as cases stratified by age group for each indicator. Age groups were aggregated into broader categories (10–29, 30–49, 50–69, 70+ years) than those used in the descriptive analyses to increase case counts, as the Farrington flexible algorithm performs more reliably when based on higher numbers of observations.

For readability, figures were restricted to 3 weeks before the start and 3 weeks after the end of the tournament, while signal detection covered the broader observation window described above. Detected overall signals were visualized over time in relation to the game days of the German national team (Supplement 3), as previous reports have described deviations in ED visits during games in the later stages of the tournament involving the host country (40). Weekday-specific analyses were conducted to describe mean relative proportions by day of the week for each indicator (Supplement 4), and time of presentation was examined to determine whether cases occurred immediately after midnight following game days.

Descriptive analyses, signal detection and data visualization were performed using programming language R (version 4.4.0) (41). The analyses were conducted using the tidyverse package (including dplyr and ggplot2) (42) and additional R packages including readxl (43), table1 (44), zoo (45), scales (46), ggpubr (47), surveillance (48) and kableExtra (49).

## 3 Results

### 3.1 Study population

A total of 41 EDs from the Emergency Department Data Registry consented to participate in this study, including two pediatric EDs. One ED was excluded from the study due to a data transmission interruption beginning on 15 March 2024, which led to missing data for the tournament and reference period. Another ED was excluded as it reported visits but did not transmit any ICD-10-GM diagnoses or CEDIS-PCL chief complaint information; therefore, none of the visits were eligible for case identification. For analyses, data from 1 January 2018 to 9 September 2024 were included, resulting in a final dataset comprising 5,602,933 visits from 39 EDs. The tournament was held in 10 cities across seven federal states: Berlin, Cologne, Dortmund, Dusseldorf, Frankfurt am Main, Gelsenkirchen, Hamburg, Leipzig, Munich and Stuttgart. Of these, four host cities were represented by a total of 10 EDs in the study (Berlin, Leipzig, Munich, Stuttgart). Overall, the 39 EDs included in the analyses were distributed across nine federal states: Baden-Wurttemberg, Bavaria, Berlin, Lower Saxony, North Rhine-Westphalia, Saxony, Saxony-Anhalt, Schleswig-Holstein and Thuringia.

### 3.2 Descriptive analyses

All three syndrome definitions consistently identified cases to capture substance use patterns in ED data throughout the entire study period and exhibited temporal variation in relative proportions of cases among eligible visits, reflecting seasonal trends and day-of-week patterns. On average, cases identified under the umbrella syndrome definition SATX accounted for 2.6% of eligible ED visits during the study period (1 January 2018 to 9 September 2024). STX cases represented 1.2%, while ATX cases accounted for 1.1% of overall visits with at least one coded diagnosis (Figure 1).

**Figure 1:**
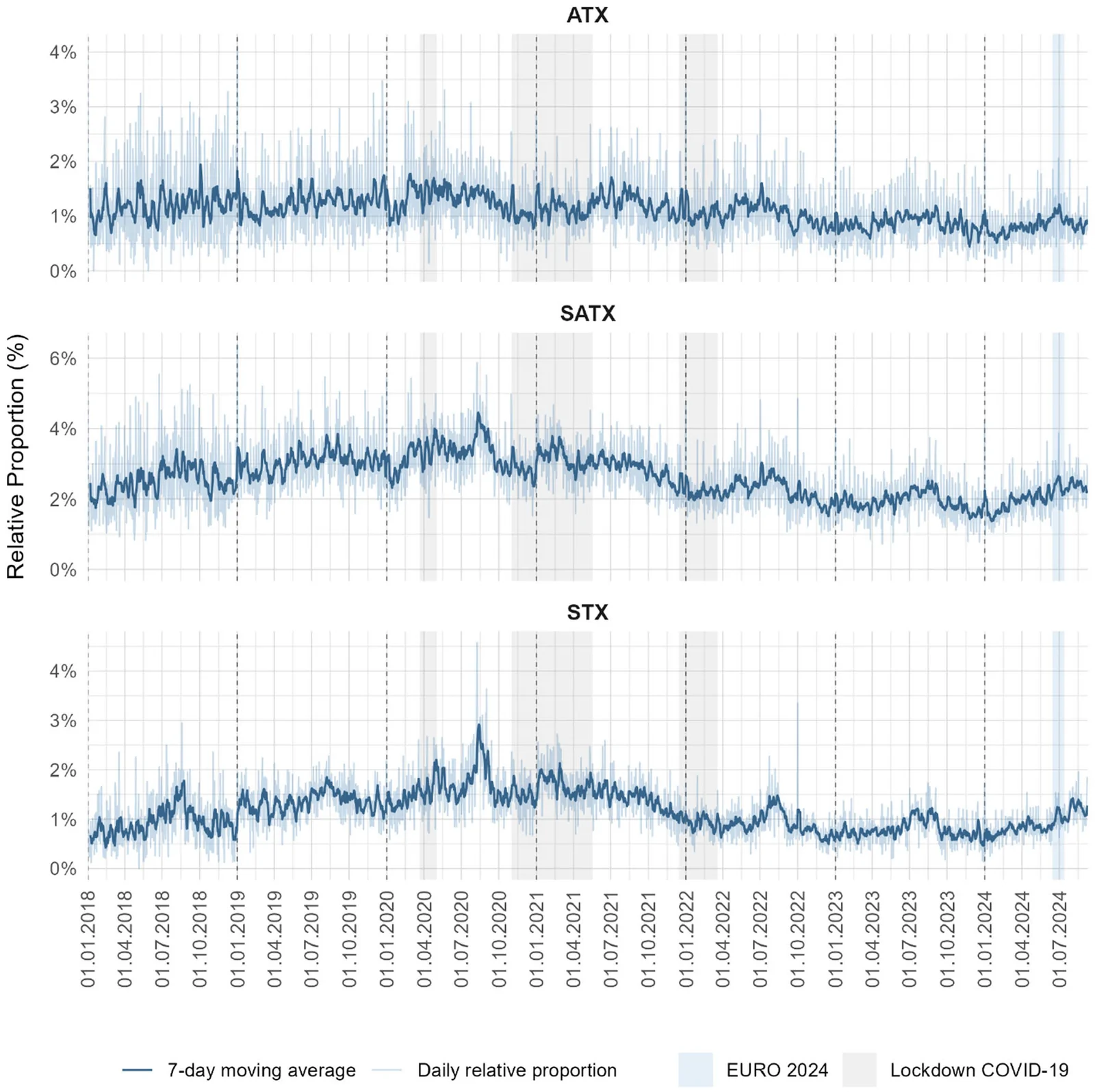
Seven-day moving average of the relative proportion of cases among eligible visits for each indicator SATX, ATX and STX.

Across the study period, day-of-week effects were more pronounced for ATX and SATX, with increasing proportions toward the end of the week. For ATX, the highest relative proportion among eligible cases was observed on Saturdays (1.7%), while the lowest occurred on Mondays (0.7%). For SATX, proportions likewise peaked on Saturdays (3.3%) and were lowest on Mondays (2.2%). In contrast, STX showed less pronounced weekday variation, with only a modest increase on Saturdays (1.3%) and the lowest proportions observed from Monday to Thursday (1.1%) (Supplement 4).

All three time series demonstrated a summer seasonality, most pronounced for STX with a peak in August of every year. Following the nationwide lockdown implemented in Germany on 22 March 2020 to contain the COVID-19 pandemic, ATX and SATX showed minor fluctuations with a slight decline in relative case numbers. In contrast, STX exhibited a persistently elevated relative proportion over the subsequent 2 years, highlighted by a notable peak in August 2020, during the summer period following the initial lockdown.

Comparing identified cases of each indicator during the tournament period to the reference period, approximately 150 additional cases were detected for SATX (Δ% = +6.7 %), 100 for ATX (Δ% = +14 %), and 50 for STX (Δ% = +6.4 %). The overall case distribution by sex, age, and triage level remained largely consistent (Table 1).

**Table 1:** Absolute case numbers and relative proportions of SATX, ATX, and STX cases stratified by sex, age group, and triage level during the tournament and reference periods, including the relative percentage change between periods.

	SATX			ATX			STX		
Characteristic	Tournament period^1^	Reference period^1^		Tournament period^1^	Reference period^1^		Tournament period^1^	Reference Period^1^	
	(*N* = 2,357)	(*N* = 2,208)	Δ%^3^	(*N* = 855)	(*N* = 750)	Δ%^3^	(*N* = 901)	(*N* = 847)	Δ%^3^
Sex									
Female	695 (31.2%)	689 (34.3%)	−9.0%	212 (25.8%)	204 (29.3%)	−11.9%	313 (37.1%)	283 (39.1%)	−5.1%
Male	1,530 (68.8%)	1,320 (65.6%)	+4.9%	611 (74.2%)	492 (70.6%)	+5.1%	531 (62.9%)	440 (60.9%)	+3.3%
Age group									
10–19	186 (8.4%)	176 (8.8%)	−4.5%	67 (8.1%)	64 (9.2%)	−12.0%	76 (9.0%)	69 (9.5%)	−5.3%
20–29	387 (17.4%)	371 (18.4%)	−5.4%	123 (14.9%)	114 (16.4%)	−9.1%	179 (21.2%)	160 (22.1%)	−4.1%
30–39	444 (20.0%)	385 (19.1%)	+4.7%	151 (18.3%)	132 (18.9%)	−3.2%	204 (24.2%)	171 (23.7%)	+2.1%
40–49	448 (20.1%)	380 (18.9%)	+6.3%	181 (22.0%)	142 (20.4%)	+7.8%	149 (17.7%)	119 (16.5%)	+7.3%
50–59	350 (15.7%)	319 (15.9%)	−1.3%	162 (19.7%)	138 (19.8%)	−0.5%	90 (10.7%)	83 (11.5%)	−7.0%
60–69	232 (10.4%)	234 (11.6%)	−10.3%	93 (11.3%)	81 (11.6%)	−2.6%	69 (8.2%)	66 (9.1%)	−9.9%
70–79	105 (4.7%)	90 (4.5%)	+4.4%	35 (4.3%)	20 (2.9%)	+48.3%	37 (4.4%)	29 (4.0%)	+10.0%
80+	73 (3.3%)	56 (2.8%)	+17.9%	11 (1.3%)	6 (0.9%)	+44.4%	40 (4.7%)	26 (3.6%)	+30.6%
Triage value^2^									
1	119 (5.3%)	111 (5.5%)	−3.6%	30 (3.6%)	32 (4.6%)	−21.7%	55 (6.5%)	38 (5.3%)	+22.6%
2	530 (23.8%)	504 (25.1%)	−5.2%	171 (20.8%)	150 (21.5%)	−3.3%	212 (25.1%)	206 (28.5%)	−11.9%
3	789 (35.5%)	715 (35.6%)	−0.3%	343 (41.7%)	278 (39.9%)	+4.5%	301 (35.7%)	243 (33.6%)	+6.3%
4	568 (25.5%)	467 (23.2%)	+9.9%	198 (24.1%)	154 (22.1%)	+9.0%	204 (24.2%)	168 (23.2%)	+4.3%
5	101 (4.5%)	114 (5.7%)	−21.1%	40 (4.9%)	42 (6.0%)	−18.3%	24 (2.8%)	27 (3.7%)	−24.3%

^1^Percent = proportion within respective period.

^2^Triage value: 1 = immediate, 2 = very urgent, 3 = urgent, 4 = standard, 5 = non-urgent.

^3^Δ% calculated as (Tournament%–Reference%)/Reference% × 100.

The most notable difference in sex distribution was found in ATX during the tournament period, with more than 74.2% of cases occurring among male patients. This pattern of higher proportions of male than female cases was also evidend for SATX and STX in both the tournament and the reference period. Additionally, a slight increase in the proportion of male cases was observed across all three indicators as reflected by the relative change comparing the tournament period to the reference period (SATX: +4.9%; ATX: +5.1%; STX: +3.3%). Sex distributions for SATX and ATX do not add up to 100% due to a small number of cases categorized as “other” in the ED data. For example, in the ATX reference period, the proportions of female (29.3%) and male (70.6%) cases sum to 99.9%, with the remaining 0.1% attributed to “other”.

During both the tournament and reference periods, the highest proportions of SATX cases were observed in the 30–39 and 40–49 age groups. For ATX, the two most affected age groups in both periods were 40–49 and 50–59 years. The STX cases were most frequently identified among younger adults, with the largest proportions occurring in the 20–29 and 30–39 age groups in both periods. Overall, these findings suggest that middle-aged adults were most affected by SATX and ATX, whereas STX showed a slight shift toward younger age groups. However, across all three indicators, older age groups exhibited higher relative changes during the tournament period, with increases of +17.9% for SATX, +44.4% for ATX, and +30.6% for STX in the 80+ age group compared to the reference period. Nonetheless, corresponding absolute case numbers in this age group were very small in both periods.

Although triage level 3 (urgent) accounted for the largest proportion of SATX cases, the most pronounced relative changes across all three indicators were observed among non-urgent cases (triage level 5), with decreases of −21.1% for SATX, −18.3% for ATX, and −24.3% for STX during the tournament period compared with the reference period. In contrast, increases were observed for triage level 4 (standard) across all indicators, most notably for SATX, which increased by +9.9%. This corresponded to approximately 100 additional SATX cases (568 during the tournament period vs. 467 during the reference period). Additionally, STX cases classified as triage level 1 (immediate) increased by +22.6% during the tournament period compared with the reference period.

### 3.3 Signal detection

To enable timely detection of deviations in identified cases for each indicator, a quasi-Poisson regression was used to calculate an expected value and a 95% PI for each day during the tournament period, as well as for the 3 weeks before and after. A total of 96 signals were detected across all indicators, including both overall signals (across all participating EDs) and signals identified within age groups. During the tournament period, 49 signals were identified for SATX, 14 for ATX, and 33 for STX. On average, three new signals occurred daily, with a maximum of nine signals detected on 29 June 2024.

Across all 39 EDs, a total of 32 overall signals were identified across the three indicators (SATX: 16, ATX: 3, STX: 13). Age-group-specific signal detection identified 64 signals in total (SATX: 33, ATX: 11, STX: 20). While application of the Farrington flexible algorithm for overall signals resulted in stable expected values and prediction intervals for all indicators, signal detection stratified by age groups appeared less robust. This was primarily due to low case numbers, particularly for ATX and STX, with some age groups lacking daily cases in the historical data, leading to signals being triggered often by only one case above the threshold.

Out of 16 overall SATX signals, nine coincided with either a Germany game day, the day following a Germany game, or the day of the finale (Figure 2). Notably, on days following Germany game days, case counts were observed in the hours after midnight, which may partly explain why some signals occurred on the next day, particularly as several games had late kick-off times (9:00 PM). A further five signals occurred on 21 June 2024, 22 June 2024, 25 June 2024, 26 June 2024, and 2 July 2024, each corresponding to game days involving national teams other than Germany. The remaining two signals were observed on Thursday, 27 June 2024 and Friday, 28 June 2024, both of which were days with no EURO 2024 games of any national team taking place. Therefore, among the nine game-free days during the tournament period, signals were detected on two days. The largest deviation from the upper threshold of the prediction interval was detected on 29 June 2024, coinciding with the Germany-Denmark game. A total of 127 cases were observed vs. 74 expected cases [95% PI: 56–93], exceeding the upper threshold by 34 additional SATX cases and accounting for 3.9% of all eligible ED visits on that day. On the day of the opening ceremony, 14 June 2024, 84 cases were observed compared to 60 expected [95% PI: 44–77]. On 14 July 2024, the day of the finale, 94 cases were observed compared with 64 expected [95% PI: 48–82]. On average, across all 16 detected signals during the tournament period, approximately 11 additional SATX cases per day were observed across all participating EDs.

**Figure 2:**
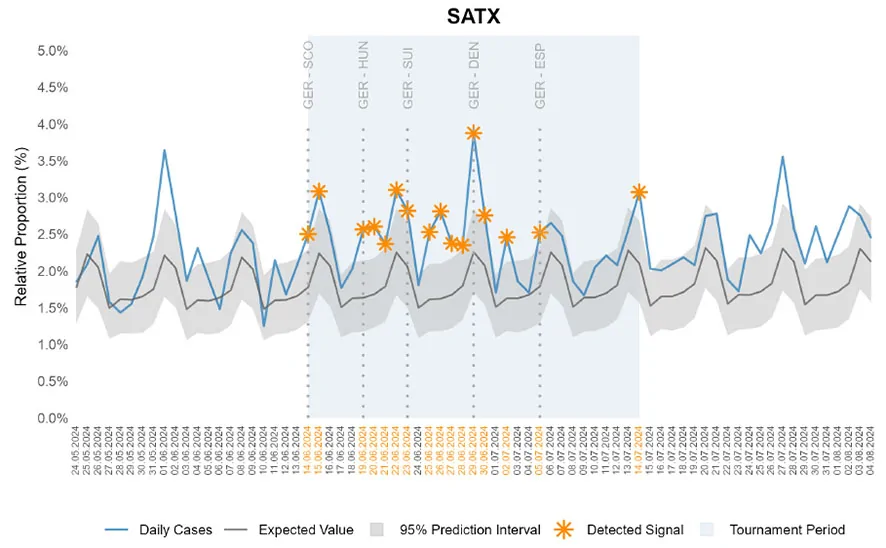
Daily detected signals for SATX cases during the EURO 24 tournament period. Games involving the host country are highlighted (dotted lines; see Supplement 3).

For ATX, a total of three overall signals were generated during the tournament period (Figure 3). Of these, two occurred on game days involving the German national team that ended in victories, and one occurred on the day following the Germany - Hungary game (GER-HUN). Observed cases during the tournament period were generally associated with higher proportions of ATX among eligible ED visits on weekends, consistent with the pattern of expected cases and the prediction interval, except for the signals detected on 19 June 2024 and 20 June 2024, which occurred on a Wednesday and a Thursday. None of these signals coincided with the opening ceremony or the finale of the tournament. Further, no signals were detected for ATX on 23 June 2024, when Germany played Switzerland in a game that ended in a draw, nor on 5 July 2024, when the German team lost to Spain and was eliminated from the tournament. For ATX, the largest deviation from the upper threshold of the prediction interval was observed on 19 June 2024, with 37 cases reported compared to the expected cases of 20 [95% PI: 12–30].

**Figure 3:**
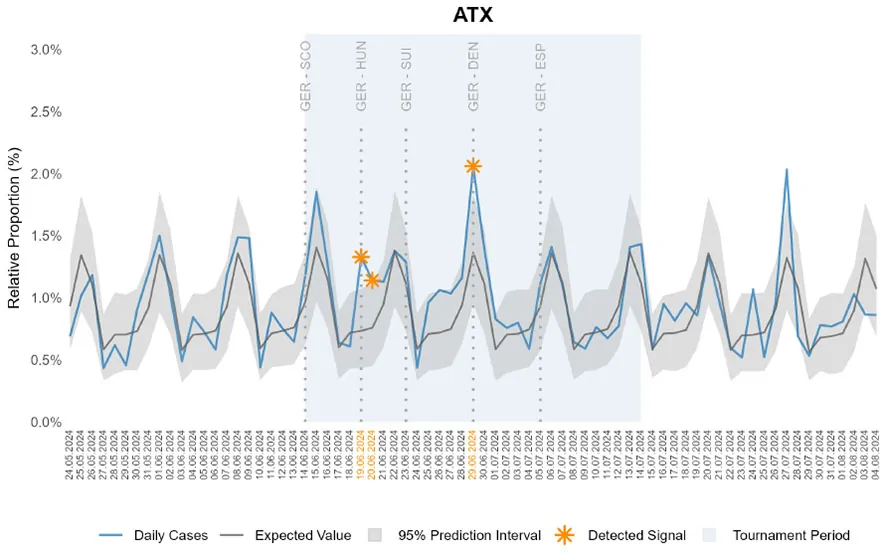
Daily detected signals for ATX cases during the EURO 24 tournament period. Games involving the host country are highlighted (dotted lines; see Supplement 3).

During the tournament period, 13 STX signals were detected. Of these, three signals coincided with Germany game days and two signals occurred on the day following a Germany game, while one was observed on the day of the finale. Only one signal was detected on a game-free day, and six additional signals were observed on days when games involving national teams other than Germany were played. Among those 13 detected signals, eight were observed on weekends (Friday - Sunday), while five signals occurred on weekdays. The largest deviation from the expected value occurred on 25 June 2024, when the observed number of cases (41) was twice as high as the expected value (20) [95% PI: 12–30], exceeding the upper threshold by 11 additional cases (Figure 4). Across all STX signals, an average of approximately five additional cases per day was observed across all participating EDs. In the 3 weeks after the tournament ended, further deviations above the upper threshold of the 95% PI were observed.

**Figure 4:**
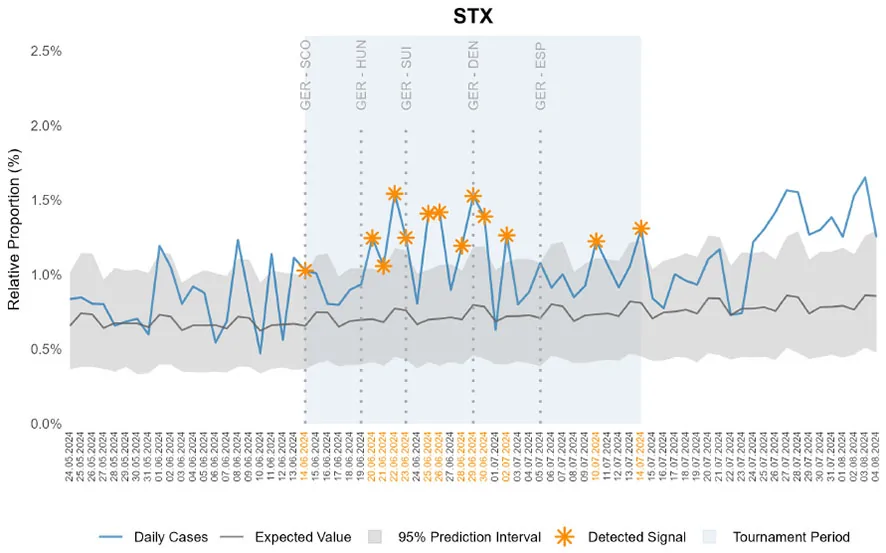
Daily detected signals for STX cases during the EURO 24 tournament period. Games involving the host country are highlighted (dotted lines; see Supplement 3).

## 4 Discussion

This study aimed to detect cases of psychotropic substance use, acute alcohol intoxication, and substance use other than alcohol during EURO 2024, examining whether these cases can be plausibly and continuously identified in ED data for real-time monitoring of trends in population health. Signal detection methods were explored to enable timely identification of deviations throughout the tournament, with the goal of supporting public health responses during MGEs in the future.

The applied syndrome definitions consistently identified substance-related ED cases over time, enabling to capture temporal patterns, including increased ED utilization on weekends and observation of summer seasonality. In particular, observed weekend effects (12, 50) of substance- and alcohol-related ED visits and the sex distribution showing a higher proportion of males (12, 15) were consistent with findings from the literature, supporting the plausibility of the identified cases for these indicators. Over the 31-day tournament period, compared to the reference period, approximately 150 additional cases were detected for SATX, 100 for ATX, and 50 for STX. In both periods, most cases occurred among middle-aged adults, particularly those aged 30–59 years for SATX and ATX.

During the EURO 2024 tournament period, signal detection revealed moderate increases in substance-related ED visits, with multiple SATX signals aligning with Germany game days and the finale. Previous studies focusing on alcohol-related ED visits across different sporting tournaments have reported significant increases, especially on game days compared with non-game days (12), which is consistent with the ATX signals detected in this study. However, during the EURO 2016 football tournament in Marseille, alcohol-related ED visits increased by 43% (25), a substantially larger effect than observed in this study, where ATX cases increased by 14% during the tournament period compared with the reference period.

Beyond overall changes observed during the tournament, potential associations between national team game outcomes (win, draw, loss) and substance-related ED visits remain largely unexplored and were not the focus of the present analysis either. However, a study from England using police data (51) reported increases in alcohol-related domestic abuse following national team victories, but not after draws or losses, and additionally identified increases on the day following game days. Although this evidence does not directly address ED utilization, it raises the question of whether game outcomes may similarly affect alcohol-related ED visits. In the present study, no ATX signals were detected following Germany's draw against Switzerland or loss to Spain during EURO 2024, whereas three ATX signals were observed on days that coincided with German victories and/or the following day. These observations are descriptive and not conclusive, reflecting temporal associations rather than causal effects of EURO 2024. Importantly, the surveillance data does not provide information on whether the identified ED visits attended the tournament or any related events. They should therefore be regarded as hypothesis-generating, pointing to a potential focus for future investigations.

Prior research has also reported increases in substance-related ED visits including alcohol during the opening ceremonies of the 2012 Olympic and Paralympic Games in London (21, 26). This pattern was similarly observed in our study, as a SATX signal was detected on the day of the EURO 2024 opening ceremony (14 June 2024) and the following day. Additionally, ED visits related to illicit drug use increased after the closing ceremony of the 2000 Olympic Games in Australia (23). In the present study, one STX signal was observed on the day of the finale as well. However, SATX and STX signals were not exclusively aligned with Germany game days or the finale, as multiple signals were also detected on other days during the tournament period, although less frequently on game-free days compared to days when other national teams played.

Importantly, these findings underscore the potential of signal detection methods to identify deviations in substance-related ED visits. At the same time, only moderate increases in absolute ED cases were observed, suggesting that no substantial additional burden was placed on participating EDs that would have required rapid public health intervention. One possible explanation for this finding is the extensive deployment of German Red Cross (DRK) medical services at stadiums and public viewing sites throughout the tournament. On-site ambulatory care aims to maximize efficiency in managing potential health hazards, minimize ambulance transports, and thereby mitigate strain on local healthcare infrastructure (52). A systematic literature review (5) reported mixed findings, with some studies suggesting that on-site health services during MGEs primarily manage low-severity cases, thereby limiting ED visits to patients with higher urgency, while other studies have reported increases in substance-related ED visits during MGEs, generally involving low-severity cases (53). In the present study, decreases in relative change during the tournament period compared with the reference period were observed for all three indicators among non-urgent triage cases (triage level 5): SATX (−21.1%), ATX (−18.3%), and STX (−24.3%). This pattern is consistent with the assumption that DRK services may have absorbed less severe cases during the tournament. However, these relative changes reflected only minor differences in absolute case numbers and may also represent routine variability in ED utilization. In contrast, increases were observed for triage level 4 (standard) across all indicators during the tournament period (SATX: +9.9%, ATX: +9.0%, STX: +4.3%). This finding contradicts the assumption of a reduction in less urgent ED visits due to on-site medical care, as more cases classified as still low urgency (triage level 4) presented during the tournament period compared with the reference period. The DRK reported providing medical care and support in 4,768 cases during EURO 2024, with 398 instances requiring patient transport. Importantly, substance-related incidents were not identified as primary causes for transport; instead, cardiovascular conditions accounted for the majority of DRK interventions (54). Notably, STX cases classified as triage level 1 (immediate) increased by +22.6% compared to the reference period, which may indicate a higher number of patients with substance use other than alcohol presenting with higher urgency.

Alongside DRK services, additional interventions implemented to mitigate health risks during the tournament period, may have contributed to less severe increases in substance-related ED visits in this study. Such interventions included reducing the alcohol concentration in beer to prevent tourists from underestimating its effect, increasing the prices of alcoholic beverages at stadiums, and providing drinking fountains to promote tap water consumption as a heat prevention measure (55). Consequently, although previous studies have reported more pronounced changes during similar events in host countries, the relatively modest changes observed during the tournament period in this study appear plausible given the extensive preventive interventions implemented in Germany. This interpretation is further supported by retail sales data on alcoholic beverages. Contrary to expectations of increased alcohol consumption associated with hosting EURO 2024, beer sales declined in June 2024 by 11.2% compared with the same month of the previous year. Notably, June 2024 recorded the lowest beer sales observed since the 1993 revision of the beer tax law (56). These reports support the finding that an anticipated surge in alcohol consumption during the tournament period may not have materialized at the national level. However, changes in travel patterns during June 2024 and July 2024 compared to the previous year varied across host cities, with the Ruhr area experiencing a more prominent increase in visitor numbers than Berlin, Hamburg or Munich (57). Consequently, the preparedness of EDs to respond to changes in ED utilization during EURO 2024 may have differed by location. While the overall findings suggest no alarming increase in healthcare demand at the national level, localized increases in patient volumes and temporary overcrowding in EDs cannot be fully ruled out. Moreover, urban areas may be better equipped to absorb short-term fluctuations in healthcare demands due to greater capacity or experience with MGEs or tourism. Additionally, already strained EDs may have been faced with substance-related visits that typically require extensive clinical resources, workforce attention and long treatment durations. However, the available data in this study did not include information on the duration patients spent in the ED (length-of-stay data), which limits the ability to fully assess potential capacity constraints in participating EDs. Such evaluations are beyond the scope of surveillance efforts and therefore the scope of the present analysis.

Other limitations should be considered when interpreting the findings of this study. In particular, the current coverage of RKI's ED surveillance system represents a major limitation for the effective monitoring of ED utilization and disease burden during MGEs. In particular, the limited regional representation of some federal states and host cities does not allow for more detailed stratification of signal detection, such as analyses focused specifically on areas surrounding match venues. Nevertheless, we did examine deviations in cases observed during tournament period compared to the reference period, stratified by those 10 participating EDs in host cities. These analyses showed no substantial differences in the distributions by sex, age group, or triage level compared with the analyses included in this study, providing no evidence that EDs closer to the venues were more affected. In addition, approximately 2,100 EURO-related events were organized across Germany, including around 940 public viewing events and numerous fan zones, which aimed at broader accessibility and engagement. These gatherings created population-wide changes in the social and environmental context during the tournament by providing additional opportunities for substance use. Consequently, while local effects in host cities may be underrepresented due to limited coverage, a nationwide surveillance approach remains meaningful, as changes in substance-related ED utilization may also have occurred in regions beyond the immediate proximity to stadiums. Due to low coverage, age-stratified signals were not further included in analyses. Improved ED coverage and longer duration of data availability may enable more robust age-specific signal detection in future applications as well.

Furthermore, case identification relies on documentation practices of the ED personnel. Heterogeneity in the completeness of diagnoses and chief complaints across participating EDs reduces the accuracy of the syndrome definitions in correctly identifying cases. Each of the three syndrome definitions used in this study generally differs in specificity, which may lead to varying degrees of underreporting or overestimation. Cases classified under the ATX definition are expected to accurately reflect true events of heavy episodic drinking, as they are based on specific ICD-10-GM coded diagnoses. However, underreporting remains likely as alcohol consumption is not explicitly recorded in the relevant chief complaint categories (CEDIS-PCL: 751 - substance misuse/intoxication; 752 -overdose/ingestion), meaning ED visits documented exclusively with these CEDIS-PCL codes without an additional ICD-10-GM diagnosis indicating alcohol use could not be identified as ATX. Similar limitations affect the STX definition, where unspecific or incomplete documentation may lead to an underreporting of cases. To address this, the broader SATX definition was developed as an umbrella category. This approach leverages routine ED data by less substance-specific case definitions, allowing the inclusion of non-specific variables and thereby reducing the risk of missing cases. However, the applied syndrome definitions may identify cases of accidental intoxication, such as overdoses in individuals with cognitive impairments (e.g., dementia), potentially leading to misclassification. It should also be noted that repeated ED visits by the same individuals may lead to an overestimation of case counts, as unique patient identifiers are unavailable due to data anonymization. Further, since EDs primarily focus on treating acute symptoms, substance intoxications may go unrecorded when they occur alongside injuries or other medical conditions. Patients may also be reluctant to disclose illicit substance use due to stigma or fear of legal consequences (58), which can lead to incomplete or inaccurate documentation, particularly affecting the identification of STX and SATX cases.

Additionally, mentioned heterogeneity in the completeness of diagnoses and chief complaints, as well as variation in the start of data transmission across participating EDs within the study population, may have affected the performance of signal detection, as the Farrington flexible algorithm relies on historical data to estimate expected values and the upper thresholds of the prediction intervals. While the algorithm adjusts for seasonality, long-term and repetitive trends such as weekend effects, the historical data used to calculate expected values included the COVID-19 pandemic, which may have affected estimation accuracy, as changes in ED utilization were reported during this time (59). To account for varying completeness of data reporting, analyses were conducted using relative proportions of cases, with denominators restricted to only eligible ED visits meeting the inclusion criteria of the respective syndrome definitions.

The performance of the prediction intervals for overall signals appeared robust across all three indicators. However, daily signal detection for STX and SATX cases during the EURO 2024 showed that observed cases remained consistently above the expected values from mid-June onwards and exceeded the upper threshold of the 95% PI in the 3 weeks following the tournament as well. These deviations may also partly reflect variations in the quality of the historical data. Additional stratification by age group then relied on very small case counts, which increased the likelihood of signal detection driven by minor increases in absolute cases that likely reflected routine data noise rather than meaningful deviations, that would not be considered relevant for continuous public health surveillance. Consequently, such stratified analyses were not included in the present study. Currently, further evaluation and refinement of both the syndrome definitions and the applied signal detection methods are planned, including validation of SATX and STX against reference data to assess their performance and inform further optimization for surveillance. In addition, it can be expected that signal detection performance will improve with expanded ED coverage and extended continuous data transmission periods, allowing the accumulation of more historical data for improved calculation of prediction intervals.

In summary, as proof of principle, our study demonstrated that rapid changes in substance-related ED visits during MGEs can be effectively monitored using the syndrome definitions and applied signal detection methods. It is, however, important to consider that observed signals cannot causally be attributed to the EURO 2024, as no information is available on the specific circumstances or context of the patients seeking ED care. The signals detected during the tournament period may also be partly influenced by seasonal factors, as changes in ED utilization were for instance shown to be associated with heat waves (60). Other concurrent events beyond EURO 2024 such as music festivals, may also have contributed to the observed patterns in substance-related ED visits. Additionally, regional differences in alcohol consumption behaviors, with the highest per capita intake reported in Eastern Germany (61), may introduce variation to the data and limit the representativeness of findings across country. This concern also applies to EDs included in this study, which may not be representative of all other EDs in Germany, particularly given that participation was based on voluntary consent.

Nevertheless, consistent and continuous data collection remains key for understanding the impact of MGEs on substance-related ED visits. Evidence in the existing literature is heterogeneous and is very limited in Germany. Therefore, this study provided a first multicentre perspective on dynamics of substance-related ED utilization at the national level and demonstrated the potential to support real-time scenarios. Importantly, this approach may inform other contexts characterized by population-wide changes in social and environmental conditions in the future, including national holidays, large-scale cultural, religious or sporting events, extreme weather conditions and periods of political changes.

## 5 Conclusion

Overall, increases in substance-related ED visits were observed during the tournament period, with daily deviations detected as signals, some of which corresponded to the German's team game days, including the opening ceremony and the finale. However, absolute case numbers underlying these daily deviations were still moderate. Accordingly, the findings rather provide reassurance that the EURO 2024 did not result in an alarming additional burden on participating EDs that would have required immediate public health intervention, while also confirming that relevant shifts in demand would have been detected if occurred. Signal detection based on these three indicators therefore represents a valuable tool for intensified syndromic surveillance during future MGEs. In the long term, expanding ED coverage would further improve the assessment of regional variations, particularly through greater inclusion of EDs in host cities.

## Data Availability

Aggregated data can be made available upon request after agreement with the AKTIN Infrastructure and Emergency Department Data Registry. The programming code is available upon request from the corresponding author. Requests to access these datasets should be directed to office@aktin.org.

## Group members of AKTIN Research Group

Caroline Grupp, Zentrale Notaufnahme, Ostalb-Klinikum-Aalen, Aalen. Bernhard Kumle, Klinik für Akut- und Notfallmedizin, Schwarzwald-Baar Klinikum Villingen-Schwennigen, Villingen-Schwennigen. Bernadett Erdmann, Zentrale Notfallaufnahme, Klinikum Wolfsburg, Wolfsburg. Conrad Grehn und André Gries und Eric Handmann, Zentrale Notaufnahme/Notaufnahmestation, Universitätsklinikum Leipzig, Leipzig. Domagoj Schunk, Interdisziplinäre Notaufnahme und Aufnahmestation sowie Interdisziplinäre Kindernotaufnahme, Universitätsklinikum Schleswig-Holstein Campus Kiel, Kiel. Christoph Lewejohann und Matthias Nürnberger, Zentrum für Notfallmedizin, Universitätsklinikum Friedrich-Schiller-Universität Jena, Jena. Philipp Kümpers, Zentrale Notaufnahme, Universitätsklinikum Münster, Münster. Stephan Arlt, Notfallzentrum Merseburg, Carl-von-Basedow-Klinikum Saalekreis gGmbH, Merseburg. Harald Dormann und Marina Karg, Zentrale Notaufnahme, Klinikum Fürth, Fürth. Martin Bergold und Thomas Henke, Zentrum für Notfallmedizin, Evangelisches Krankenhaus Oldenburg, Oldenburg. Bernd Ulrich, Notfallaufnahme & Kindernotaufnahme, Klinikum Oldenburg, Oldenburg. Christian Reich und Andreas Hauf, Zentrale Notaufnahme, Sankt Joseph Krankenhaus Berlin-Tempelhof, Berlin. Thomas Peschel, Zentrale Notaufnahme, Elblandklinikum Radebeul, Radebeul. Thomas Peschel, Notfallzentrum, Elblandklinikum Meißen, Meißen. Thomas Peschel, Interdisziplinäre Notaufnahme, Elblandklinikum Riesa, Riesa. Kirsten Habbinga, Notaufnahme, Pius Hospital Medizinischer Campus Universität Oldenburg, Oldenburg. Norbert Pfeufer, Zentrale Notaufnahme, Kliniken Landkreis Heidenheim, Heidenheim an der Brenz Clemens Kill und Randi Katrin Manegold, Zentrum für Notfallmedizin, Universitätsmedizin Essen, Essen. Heike Höger-Schmidt und Thomas Baitz, Zentrale Notaufnahme, Klinikum Chemnitz, Chemnitz. Jörg Brokmann und Carsten Mach, Zentrale Notaufnahme, Uniklinik RWTH Aachen, Aachen. Rupert Grashey, Notfallklinik, Klinikum Memmingen, Memmingen. Martin Möckel und Ralf Offermann und Anna Slagman, Charité Notfallzentrum Mitte, Charité, Berlin. Rajan Somasundaram und Eike-Christian Kühn, Zentrale Notaufnahme, Charité Universitätsmedizin Berlin Campus Benjamin Franklin, Berlin. Martin Möckel und Ralf Offermann und Anna Slagman, Zentrale Notaufnahme, Charité, Berlin. Tobias Hofmann und Susanne Drynda, Zentrale Notaufnahme, Universitätsmedizin Magdeburg, Magdeburg. Matthias Klein, Zentrale Notaufnahme, Klinikum Großhadern Ludwig-Maximilians-Universität München, München. Matthias Klein, Zentrale Notaufnahme, Klinikum Innenstadt Ludwig-Maximilians-Universität München, München. Thomas Händl, Zentrale Notaufnahme, Klinikum Garmisch-Partenkirchen, Garmisch-Partenkirchen. Christian Wrede, Interdisziplinäres Notfallzentrum, Helios-Klinikum Berlin Buch, Berlin. Markus Wehler und Jürgen Neubauer, Zentrale Notaufnahme, Universitätsklinikum Augsburg, Augsburg. Christian Dumpies, Zentrale Notaufnahme, BG Klinikum Bergmannstrost Halle, Halle. Johannes Jubitz und Fabienne Jacob, Zentrale Notaufnahme, Paracelsus Kinik, Henstedt-Ulzburg Sabine Blaschke-Steinbrecher und Katrin Esslinger, Zentrale Notaufnahme, Universitätsmedizin Göttingen, Göttingen. Sebastian Wolfrum, Interdisziplinäre Notaufnahme, Universitätsklinikum Schleswig-Holstein, Lübeck. Tobias Schilling, Interdisziplinäre Notaufnahme, Klinikum Stuttgart, Stuttgart. Friedrich Reichert, Pädiatrische Interdisziplinäre Notaufnahme, Klinikum Stuttgart, Stuttgart. Jacqueline Bauer, Kindernotfallambulanz, Klinikum Wolfsburg, Wolfsburg. Stephan Höft und Stephanie Matis und Sabrina Heinrich, Zentrale Notaufnahme, Städtisches Klinikum Braunschweig, Braunschweig. Markus Zimmermann und Frank Hanses, Interdisziplinäre Notaufnahme, Universitätsklinikum Regensburg, Regensburg. Volker Burst, Zentrale Notaufnahme, Uniklinik Köln, Köln. Hans-Jörg Busch und Felix P. Hans, Zentrum für Notfall- und Rettungsmedizin Universitäts-Notfallzentrum, Universitätsklinik Freiburg, Freiburg. Jonas Bienzeisler, Institut für medizinische Informatik, Uniklinik RWTH Aachen. Hauke Heidemeyer, Institut für medizinische Informatik, Uniklinik RWTH Aachen. Alexander Kombeiz, Institut für medizinische Informatik, Uniklinik RWTH Aachen. Raphael Majeed, Institut für medizinische Informatik, Uniklinik RWTH Aachen. Rainer RÖhrig, Institut für medizinische Informatik, Uniklinik RWTH Aachen. Susanne Drynda, IPHAM und Medizinische Fakultät, Universitätsmedizin Magdeburg. Saskia Gieraths, IPHAM und Medizinische Fakultät, Universitätsmedizin Magdeburg. Ronny Otto, IPHAM und Medizinische Fakultät, Universitätsmedizin Magdeburg. Wiebke Schirrmeister, IPHAM und Medizinische Fakultät, Universitätsmedizin Magdeburg. Felix Walcher, IPHAM und Medizinische Fakultät, Universitätsmedizin Magdeburg.
