# Supplementary material for: Signal detection of substance-related emergency department visits during UEFA EURO 2024 in Germany: enhancing public health surveillance for future mass gatherings
Source: Front Public Health. 2026 Oct 5;14:1876317. doi: 10.3389/fpubh.2026.1876317 (PMC13677532; doi:10.3389/fpubh.2026.1876317)
Supplement: Supplementary file 1 [file Table_1.docx]

**Supplementary Material**

**Supplement 1.** Three developed syndrome definitions**:** Diagnoses, chief complaints and age requirement included for case identification of each indicator

| **Indicator** | **Abbreviation** | **ICD-10-GM Diagnosis** (certainty G, V, NA)**¹** | **Chief Complaint as CEDIS-PCL** | **Age** |
| --- | --- | --- | --- | --- |
| **Substance Intoxication** All psychotropic substances | SATX | **F10-F19** Mental and behavioral disorders due to psychoactive substance use | **751** Substance abuse/ Intoxication | >= 10 years |
|  |  | **T36-T50** Poisoning by drugs, medicaments and biological substances | **752** Ingestion of an overdose |  |
|  |  | **T51-T65** Toxic effects of substances chiefly nonmedicinal as to source |  |  |
| **Acute Alcohol Intoxication** | ATX | **F10** Mental and behavioral disorders due to use of alcohol |  | >= 10 years |
|  |  | **T51.9** Toxic effect of alcohol, unspecified |  |  |
| **Substance Intoxications Psychotropic** substances other than alcohol | STX | **F11-F19** Mental and behavioral disorders due to psychoactive substance use |  | >= 10 years |
|  |  | **T36-T50** Poisoning by drugs, medicaments and biological substances |  |  |
|  |  | **T52-T65** Toxic effects of substances chiefly nonmedicinal as to source |  |  |
| ¹*certainty G, V, NA* = ICD-10-GM diagnosis certainty: G = confirmed diagnosis (gesichert), V = suspected diagnosis (Verdacht auf), NA = not available | | | | |

**Supplement 2.** Detected signals during the tournament period by indicator, including signal date, observed and expected cases, lower and upper threshold of 95% prediction interval, the deviation between observed cases and the upper threshold, and daily ED attendance

| **Date of Signal** | **Observed Cases** | **Expected Cases** | **95% Prediction Interval** | **Deviation to Upper Threshold¹** | **ED Attendance²** |
| --- | --- | --- | --- | --- | --- |
| **SATX** | | | | | |
| 14 June 2024 | 84 | 60 | 44 - 77 | + 7 | 3,358 |
| 15 June 2024 | 93 | 68 | 51 - 86 | + 7 | 3,015 |
| 19 June 2024 | 85 | 54 | 39 - 71 | + 14 | 3,313 |
| 20 June 2024 | 91 | 59 | 43 - 76 | + 15 | 3,496 |
| 21 June 2024 | 83 | 63 | 46 - 81 | + 2 | 3,507 |
| 22 June 2024 | 96 | 70 | 52 - 88 | + 8 | 3,095 |
| 23 June 2024 | 79 | 58 | 42 - 75 | + 4 | 2,804 |
| 25 June 2024 | 88 | 56 | 41 - 73 | + 15 | 3,480 |
| 26 June 2024 | 95 | 55 | 40 - 71 | + 24 | 3,379 |
| 27 June 2024 | 82 | 58 | 42 - 75 | + 7 | 3,453 |
| 28 June 2024 | 86 | 66 | 49 - 84 | + 7 | 3,661 |
| 29 June 2024 | 127 | 74 | 56 - 93 | + 34 | 3,276 |
| 30 June 2024 | 84 | 63 | 47 - 81 | + 3 | 3,048 |
| 02 July 2024 | 81 | 54 | 39 - 70 | + 11 | 3,296 |
| 05 July 2024 | 80 | 57 | 41 - 74 | + 6 | 3,171 |
| 14 July 2024 | 94 | 64 | 48 - 82 | + 12 | 3,059 |
|  |  |  |  |  |  |
| **ATX** | | | | | |
| 19 June 2024 | 37 | 20 | 12 - 30 | + 7 | 2,786 |
| 20 June 2024 | 33 | 22 | 13 - 32 | + 1 | 2,894 |
| 29 June 2024 | 54 | 36 | 25 - 48 | + 6 | 2,621 |
|  |  |  |  |  |  |
| **STX** | | | | | |
| 14 June 2024 | 28 | 18 | 10 - 27 | + 1 | 2,730 |
| 20 June 2024 | 36 | 20 | 12 - 30 | + 6 | 2,894 |
| 21 June 2024 | 30 | 19 | 11 - 29 | + 1 | 2,840 |
| 22 June 2024 | 38 | 19 | 11 - 29 | + 9 | 2,466 |
| 23 June 2024 | 29 | 18 | 10 - 27 | + 2 | 2,327 |
| 25 June 2024 | 41 | 20 | 12 - 30 | + 11 | 2,913 |
| 26 June 2024 | 40 | 20 | 11 - 30 | + 10 | 2,823 |
| 28 June 2024 | 36 | 21 | 12 - 31 | + 5 | 3,019 |
| 29 June 2024 | 40 | 21 | 12 - 31 | + 9 | 2,621 |
| 30 June 2024 | 35 | 20 | 11 - 30 | + 5 | 2,521 |
| 02 July 2024 | 35 | 20 | 11 - 30 | + 5 | 2,773 |
| 10 July 2024 | 32 | 19 | 11 - 29 | + 3 | 2,618 |
| 14 July 2024 | 32 | 20 | 11 - 30 | + 2 | 2,447 |
| ¹*Deviation to Upper Threshold* = Deviations of observed cases to upper threshold of 95% Prediction Interval | | | | | |
| ²*ED Attendance =* total number of eligible ED visits for the indicator on that day | | | | | |

**Supplement 3.** UEFA EURO 2024 – Game Days involving the German National Team, including tournament round, kick-off time and final result

| **Tournament Round** | **Game Day** | **Kick-off** | **Game** | **Result** | **Abbreviation** |
| --- | --- | --- | --- | --- | --- |
|  |  |  |  |  |  |
| Group Phase | 14 June 2024 | 9:00 PM | Germany - Scotland | 5:1 | GER - SCO |
| Group Phase | 19 June 2024 | 6:00 PM | Germany - Hungary | 2:0 | GER - HUN |
| Group Phase | 23 June 2024 | 9:00 PM | Germany - Switzerland | 1:1 | GER - SUI |
| Round of 16 | 29 June 2024 | 9:00 PM | Germany - Denmark | 2:0 | GER - DEN |
| Quarter-final | 05 July 2024 | 6:00 PM | Germany - Spain | 1:2 (*a.e.t.*)¹, *elim*² | GER - ESP |
|  |  |  |  |  |  |
| ¹*a.e.t.* = after extra time | | | | | |
| ²*elim* = eliminated from the tournament | | | | | |

**Supplement 4.** Mean daily relative proportions of ATX, SATX, and STX cases among eligible ED visits stratified by day of the week

| **Day-of-week effects for ATX, SATX, and STX** | | | |
| --- | --- | --- | --- |
|  | **ATX (%)** | **SATX (%)** | **STX (%)** |
| **Monday** | 0.7% | 2.2% | 1.1% |
| **Tuesday** | 0.9% | 2.4% | 1.1% |
| **Wednesday** | 0.9% | 2.4% | 1.1% |
| **Thursday** | 0.9% | 2.4% | 1.1% |
| **Friday** | 1.1% | 2.6% | 1.1% |
| **Saturday** | 1.7% | 3.3% | 1.3% |
| **Sunday** | 1.5% | 3.1% | 1.2% |
